# Negative pressure irrigation and endoscopic necrosectomy through man-made sinus tract in infected necrotizing pancreatitis: a technical report

**DOI:** 10.1186/s12893-016-0190-x

**Published:** 2016-11-10

**Authors:** Zhihui Tong, Lu Ke, Baiqiang Li, Gang Li, Jing Zhou, Xiao Shen, Weiqin Li, Ning Li, Jieshou Li

**Affiliations:** 1 SICU, Department of General Surgery, Jinling Hospital, Nanjing University School of Medicine, Nanjing, 210002 People’s Republic of China; 2 Department of General Surgery, Jinling Hospital, Nanjing University School of Medicine, Nanjing, 210002 People’s Republic of China; 3 Department of SICU, Research Institute of General Surgery, Jinling Hospital, 305 East Zhongshan Road, Nanjing, 210002 Jiangsu Province China

**Keywords:** Infected pancreatic necrosis, Negative pressure irrigation, Endoscopic necrosectomy, Percutaneous catheter drainage

## Abstract

**Background:**

In recent years, a step-up approach based on minimally invasive techniques was recommended by latest guidelines as initial invasive treatment for infected pancreatic necrosis (IPN). In this study, we aimed to describe a novel step-up approach for treating IPN consisting of four steps including negative pressure irrigation (NPI) and endoscopic necrosectomy (ED) as a bridge between percutaneous catheter drainage (PCD) and open necrosectomy

**Methods:**

A retrospective review of a prospectively collected internal database of patients with a diagnosis of IPN between Jan, 2012 to Dec, 2012 at a single institution was performed. All patients underwent the same drainage strategy including four steps: PCD, NPI, ED and open necrosectomy. The demographic characteristics and clinical outcomes of study patients were analyzed.

**Results:**

A total of 71 consecutive patients (48 males and 23 females) were included in the analysis. No significant procedure-related complication was observed and the overall mortality was +21.1 % (15 of 71 patients). Seven different strategies like PCD+ NPI, PCD+NPI+ED, PCD+open necrosectomy, etcetera, were applied in study patients and a half of them received PCD alone. In general, each patient underwent a median of 2 drainage procedures and the median total drainage duration was 11 days (interquartile range, 6–21days).

**Conclusions:**

This four-step approach is effective in treating IPN and adds no extra risk to patients when compared with other latest step-up strategies. The two novel techniques (NPI and ED) could offer distinct clinical benefits without posing unanticipated risks inherent to the procedures.

**Electronic supplementary material:**

The online version of this article (doi:10.1186/s12893-016-0190-x) contains supplementary material, which is available to authorized users.

## Background

Secondary infection of pancreatic necrosis (IPN), either pancreatic or peripancreatic, has been proved to be one of the most important determinants of severity in patients with acute necrotizing pancreatitis [1]. When compared with patients with sterile necrosis, patients with IPN suffered substantial increase in mortality ranging from 14 to 69 % due to sepsis and multiple organ failure, despite advances in critical care and antibiotics [2]. Traditionally, primary open necrosectomy has long been center of treatment in IPN patients [3], but in recent years, a step-up approach based on minimally invasive techniques was recommended by latest guidelines as initial invasive treatment [4]. In previous studies, percutaneous catheter drainage (PCD) is the cornerstone of step-up approaches and open necrosectomy always the last choice for those who did not respond to minimally invasive treatment [3]. However, techniques using either endoscope or laparoscope applied between PCD and open necrosectomy vary in different studies [5–8] and the optimal choice remains unknown.

In the present study, we aimed to describe both the technical and clinical aspects of a new step-up approach for treating IPN consisting of four steps including negative pressure irrigation (NPI) and endoscopic necrosectomy (ED) as a bridge between PCD and open necrosectomy. By evaluating its feasibility and safety, we aimed to establish a framework for further studies comparing clinical effectiveness of currently available minimally invasive strategies.

## Methods

Using an prospectively collected internal database, a retrospective review on all patients with a diagnosis of IPN between Jan, 2012 and Dec, 2012 at the Jinling Hospital, Nanjing University was performed. Study procedures were approved by the Jinling Hospital Institutional Review Board. The inclusion criteria included: 1) diagnosed with AP based on the Atlanta Criteria [9]; 2) age between 18 and 70 years old; 3) confirmation of IPN when one or more of the following was present: gas bubbles within pancreatic necrosis seen on Computed Tomography (CT); a positive culture obtained by fine-needle aspiration or during the first drainage and/or operative necrosectomy [1]. Patients were excluded if 1) they were pregnant; 2) they had received operative necrosectomy in other hospitals during the current episode of AP; 3) they had received abdominal surgery before IPN was present due to abdominal compartment syndrome (ACS), perforation of a visceral organ, bleeding, etc.; 4) treatment strategy was not completed due to non-medical reasons. All the patients initially received standard medical treatment according to the guidelines when IPN was not clinically diagnosed [10, 11]. Organ failure was managed with organ-specific treatment if needed, including mechanical ventilation, continuous renal replacement therapy (CRRT), vasoactive agents, invasive hemodynamic monitor (Picco2), etc.

### Definitions

The criteria for organ dysfunction were described for 3 organ systems based on recently published international consensus [1, 12]: cardiovascular (need for inotropic agent), renal (creatinine ≥171 μmol/L), and respiratory (PaO2/FiO2 ≤ 300 mmHg). Persistent organ failure was defined as organ failure in the same organ system for 48 h or more. Sepsis and septic shock was diagnosed according to SSC 2012 [13]. Gastrointestinal fistula was diagnosed when either small- or large-bowel contents were discharged from a drain or from the surgical wound. New-onset complication was defined as a complication not present at any time during the 24 h before first intervention. The severities of patients were classified at discharge or hospital death by the criteria of both the Revised Atlanta Classification (RAC) and the Determinant-based Classification (DBC) [1, 12].

### The minimally invasive approach

**Fig. 1:**
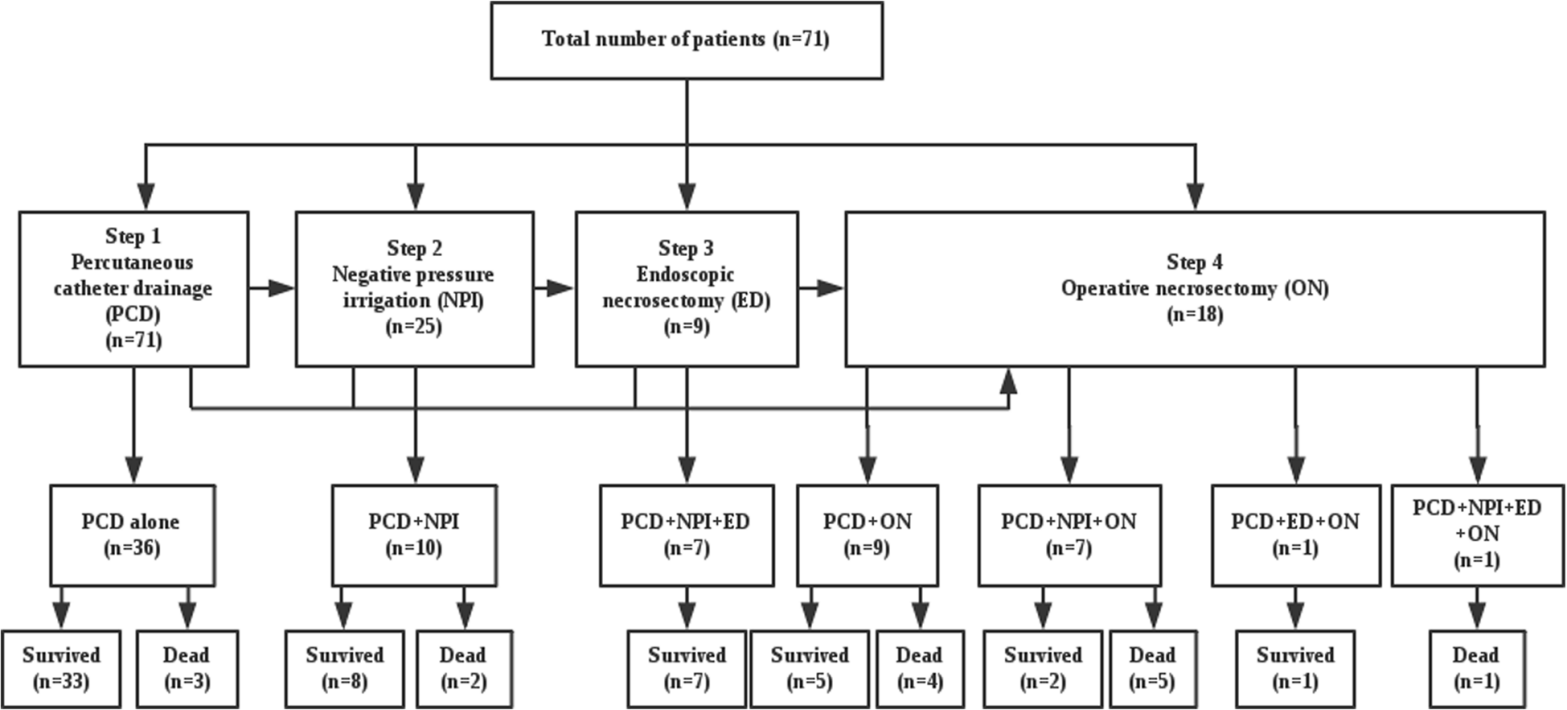
Treatment and outcome of the enrolment patients

**Fig. 2:**
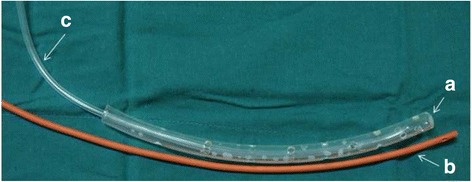
Sketch map for double catheterization cannula, which is made of 3 parts. Part *a* is a plastic dead-end tube with a diameter between 24F and 30F. There are 14–30 side apertures along the tube according to the length of the tube and the diameter of each side aperture is 5 mm. Part *b* is a 12F urinary catheter for continuous infusion of irrigation fluid. Part *c* is a plastic drainage tube inside part and it is used for continuous negative pressure drainage. The diameter of Part *c* is about half of Part *a*

The drainage strategy includes four procedures (Fig. 1): PCD, NPI, ED trough man-made sinus tract and operative necrosectomy (ON). Image-guided PCD was well described in previous studies and was also considered as the first choice in this study, the route could be through the retroperitoneum or the peritoneum depending on the location of IPN and adjacent organs [7, 14]. When the following criteria was met: 1) clinical improvement (improved organ dysfunction including circulatory, respiratory and renal, at least 10 % drop of APACHE [Acute Physiology and Chronic Health Evaluation] II score) could not be achieved through PCD alone in 3 days after procedure and CT results showed the drain was adequate, 2) mean CT density of necrotic tissue ≥30Hu or 3) suspicious or diagnosed gastrointestinal fistula, NPI would be applied as the first intervention or in addition to the drain catheters already existed and followed by ED when necessary (through the sinus tract created by NPI) before consideration of ON. NPI was implemented using “double catheterization cannula” (Fig. 2) which enables continuous irrigation of the cavity.

During the minimally invasive treatment, if patients presented one or more of 1) ACS developed and non-operative measure failed; 2) abdominal bleeding can not be controlled by conservative treatment; 3) diagnosed gastrointestinal fistula can not be well drainaged (judged by the treating physician); 4) progression of septic shock; 5) clinical improvement could not be achieved after 3 times of repeated ED. open necrosecotomy would be arranged to avoid life-threatening complications and facilitate the drainage process. Moreover, at whatever stage, return to one or more of the previous steps was allowed (e.g. patients already received ON was allowed to receive postoperative PCD as additional drainage).

**Fig. 3:**
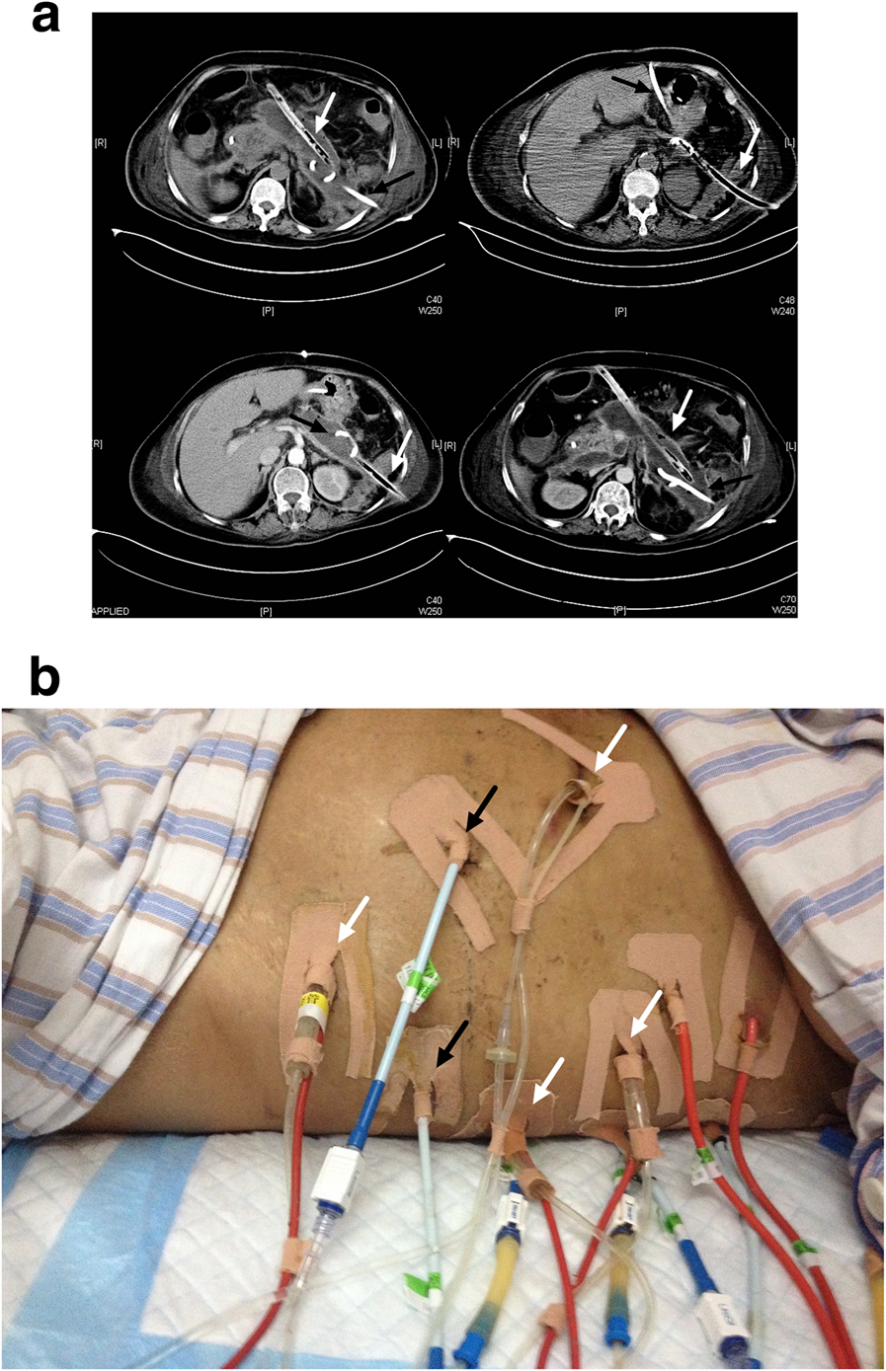
**a** Samples of “drainage system” (pig-tail catheter and double catheterization cannula within the same necrosis cavity for continuous irrigation); **b** A patient with multiple drainage catheters and double catheterization cannulas, namely, multiple “drainage systems”. The *black arrows* indicate pig-tail catheters and the *white arrows* indicate double catheterization cannulas

The “double catheterization cannula” was made of a 24–30F tube for continuous negative pressure drainage and a 12F urethral catheter for continuous infusion (see the operating mechanism of this tube in the Additional file 1, similar instrument was also described in previous literature [15]). The diameter of the hole around the tube is 5 mm and the number of the hole is 14–30 depending on the length of the tube. This cannula could be placed mini-invasively under the guidance of CT or during the operation and the route could be either peritoneal or retroperitoneal. Briefly, after the access to the necrotic cavity was obtained with a 18G hollow needle (150 mm long), a guide wire was placed into the cavity and CT scan was repeated to confirm the puncture route. Then the tract was dilated to 28F using serial renal dilators over the guide wire and the catheter was then inserted. The catheters were routinely changed every week to maximize the effect of continuous drainage. In patients received NPI, PCD would usually be additionally applied and the route would be delicately designed to construct a “drainage system” (Fig. 3) in the cavity which could potentially facilitate the drainage process.

ED was performed using electronic gastroscope (30F) through the sinus tract created by the “double catheterization cannulas” and a snare was used to drag out massive bulk of necrotic tissue (see the videos in Additional files 2 and 3) that could hardly be drained by NPI and PCD. ED can be repeated whenever deemed to be necessary, and NPI would be continuously applied in the same port during the intervals between EDs. The ON was similar to previous reports, briefly, a laparotomy through a bilateral subcostal incision was performed and several “double catheterization cannulas” or drains were inserted for postoperative lavage. All interventions were performed by the same team who were experienced in pancreatic surgery and also PCD and NPI therapy. EN procedures were performed by two experienced endoscopists who were well trained for this intervention.Additional file 2: Movie S1 Video one of endoscopic necrosectomy. Endoscopic necrosectomy was performed using electronic gastroscope (30F) through the sinus tract created by the “double catheterization cannulas” and a snare was used to drag out massive bulk of necrotic tissue.
Additional file 3: Movie S2 Video two of endoscopic necrosectomy. Endoscopic necrosectomy was performed using electronic gastroscope (30F) through the sinus tract created by the “double catheterization cannulas” and a snare was used to drag out massive bulk of necrotic tissue.


### Data collection

Demographic data including age, sex, etiology, APACHE II score, Sequential Organ Failure Assessment (SOFA) score, interval between symptom onset and admission of all study patients were recorded on admission. Outcome assessment included a composite of clinical metric to evaluate the feasibility and safety of the four-step approach such as mortality and morbidity, length of hospital and intensive care unit (ICU) stay, total treatment duration, treatment strategy for each patient, economical cost, etc. Contrast enhanced CT (CECT) was performed after admission, before discharge and on demand of the treating physician, and the CT severity index was assessed according to Balthazar’s CT score [16]. All patients were followed up for at least 3 months after discharge. The clinical characteristics of patients admitted from Jan to Jun and from Jul to Dec were compared.

### Statistically analysis

Continuous variables were expressed as medians (interquartile ranges) due to the limited sample size and the variability of the study patients. Categoric variables were described in absolute numbers and in percentages.

## Results

During the study period, a total of 71 patients underwent the minimally invasive procedure were included in the analysis. No significant treatment-related complication was observed and the overall mortality was 21.1 % (15 of 71 patients). Most patients (11 of 15) died of uncontrolled pancreatic infection with associated organ dysfunction, 3 patients died of recurrent major abdominal bleeding and another elderly patient (76 years old) died of respiratory dysfunction due to respiratory tract infection and co-existing chronic obstructive pulmonary disease (COPD). It is notable that the mortality in patients received operation (10 of 18 patients, 56 %) was significantly higher than the other patients.

### Demographics

The demographic characteristics of all 71 patients were shown in Table 1. Most patients in this study suffered the most severe type of AP (critical AP in DBC and severe AP in RAC) and the CT severity index (CTSI) score was also extremely high. Moreover, as 90 % of the study patients were transferred from other hospitals, the median time from onset of AP to admission were 23 days (interquartile range, 6–53 days). Although our patients did not show very high APACHE II score and SOFA score (Table 1), organ dysfunction at admission was very common in study population and respiratory dysfunction could be seen in more than half of the patients (71.8 %). In addition, more than a fourth of patients were admitted with existing sepsis (19 of 71, 26.7 %).

**Table 1:** Demographic data and clinical characteristics

Demographic and clinical variables (*n* = 71)	
Age (years)	45 (35 to 53)
Gender	48 males/23 females
Etiology	34 Biliary origin
	9 Alcohol abuse
	24 Hyperlipidemia
	4 Idiopathic
APACHE II score at admission	10 (7 to 15)
SOFA score at admission	3 (1 to 6)
CT severity index	10 (8–10)
Revised Atlanta Classification	Moderate AP 23 (32.4 %)
	Severe AP 48 (67.6 %)
Determinant-based Classification	Severe AP 23 (32.4 %)
	Critical AP 48 (67.6 %)
Onset of symptom to admission (days)	23 (6 to 53)
Tertiary referral	64 (90.1 %)
Organ dysfunction at admission	Respiratory 51 (71.8 %)
	Renal 21 (29.6 %)
	Cardiovascular 12 (16.9 %)
Sepsis at admission	19 (26.7 %)

### Feasibility metrics

As shown in Table 2, a total of 7 different strategies were applied in our patients and a half of the study patients received PCD only. Other commonly used strategies include PCD + NPI, PCD+ ON, PCD + NPI + ED and PCD+ NPI +ON and only one patients underwent all four steps. In general, a median of 2 drainage procedures were applied for each patient and the median total drainage duration was 11 days (interquartile range, 6–21days). About one fourth of all patients received operative necrosectomy and the mortality in these patients was noticeably high (10 of 18 patients, 56 %). The indications for the first operation included: 1) dissatisfactory drainage by minimally invasive measures and no clinical improvement (6 patients) 2) major abdominal bleeding (3 patients) 3) progress of septic complications like multiple organ dysfunction (6 patients); 4) operational enterostomy and drainage for intestinal or colonic fistulas that can not be well drainage with minimally invasive interventions (3 patients). Reoperation was rare (3 patients) and abdominal bleeding was the only reason for all cases.

**Table 2:** Metrics for feasibility

Metrics for feasibility (*n* = 71)	
Treatment approach	PCD alone 36 (50.1 %)
	PCD+NPI 10 (14.1 %)
	PCD+ON 9 (12.7 %)
	PCD + NPI + ED 7 (9.9 %)
	PCD+ NPI +ON 7 (9.9 %)
	PCD+ ED +ON 1 (1.4 %)
	PCD+NPI+ED+ON 1 (1.4 %)
Times of PCD in patients received PCD (*n* = 71)	2 (1 to 3)
No. of drainage catherters in patients received PCD (*n* = 71)	3 (2–4)
Times of NPI in patients received NPI (*n* = 25)	2 (1 to 3)
No. of NPI in patients received NPI (*n* = 25)	2 (1 to 3)
Times of ED in patients received ED (*n* = 9)	2 (2 to 4)
Patients needing operative intervention (%)	18 (25.4 %)
Patients needing reoperation (%)	3 (4.2 %)
Patients needing readmission (%)	5 (7.0 %)
Total no. of drainage procedures per patient	2 (2 to 4)
Total drainage duration (day)	11 (6–21)

For PCD, all patients received PCD either as the initial step of drainage or as a supplement and the median times and numbers of catheters placed were shown in Table 2. Similar to PCD, repeated NPI tube placement was quite common, and most patients received more than one “double catheterization cannula” for continuous irrigation. Moreover, ED was also usually applied in a repeated manner with a median of 2 times (interquartile range, 2–4 times)

### Clinical outcome and safety metrics

As shown in Table 3, the median hospital duration of our cohort was significantly long with a median of 41 day (interquartile range, 23–61 days), as well as the ICU duration. During the drainage process, new-onset organ dysfunction was seldom seen and new-onset cardiovascular dysfunction was the one with highest incidence (6 of 71 patients). In contrast, gastrointestinal fistula (colonic and duodenal for the most), pancreatic fistula and intra-abdominal bleeding were the three most commonly seen complications (Table 3). Most patients with gastrointestinal fistula were managed non-operatively (laparotomic neostomy was done in only 5 patients) and topical irrigation around the fistula site was the major intervention. According to our 6-month follow-up data, pancreatic fistula was the most common long-term complication in this cohort, incision hernia also developed in one patient.

**Table 3:** Metrics for safety and clinical outcome

Clinical outcome measures	
Mortality (%)	15 (21.1 %)
New-onset organ dysfunction	Cardiovascular 6 (8.4 %)
	Respiratory 1 (1.4 %)
	Renal 5 (7.0 %)
New-onset Sepsis	10 (14.1 %)
Gastrointestinal fistula	17 (23.9 %)
	Colonic alone 7 (9.9 %)
	Duodenal alone 5 (7.0 %)
	Jejunal or gastric alone 1 (1.4 %)
	Multiple 4 (5.6)
Pancreatic fistula	14 (19.7 %)
Chylous fistula	3 (4.2 %)
Intra-abdominal bleeding	11 (15.5 %)
Portal venous system thrombosis	3 (4.2 %)
Positive culture result for fungi	12 (16.9 %)
Gastric outlet obstruction	2 (2.8 %)
Hospital duration (day)	41 (23–61)
ICU duration (day)	17 (7–43)
Total cost (10,000 rmb)	18.2 (8.6–32.4)

Regarding other complications, none of the study patients suffered internal bleeding during the procedure of NPI. Two patients (8.0 %) developed severe abdominal bleeding during the period of NPI drainage and required interventional embolization. According to the DSA results, the bleeding events were more likely to be caused by continuous corrosion due to infected pancreatic necrosis rather than the NPI instrument, as the bleeding site is far away from the NPI tube. All bleeding events were retroperitoneal and operation was applied in 8 case in which bleeding could not be stopped by interventional embolization. Moreover, 10 patients developed new-onset sepsis or septic shock during treatment and only 3 of them were reversed. Other less common complications include chylous fistula, portal venous system thrombosis and gastric outlet obstruction and all of which showed an incidence rate less than 5 %.

## Discussion

As minimally invasive approach became the mainstream for treating IPN in recent years, we developed a new drainage protocol combining three minimally invasive techniques and operation together. With this novel four-step approach, the overall mortality in our series was 21.1 %, which is comparable to that reported in previous series [7, 17], despite that most of our patients were deemed as the most severe type of AP according to latest classifications [12, 13]. The incidence of major complications such as intra-abdominal bleeding, enterocutaneous fistula, etc. also did not dramatically differ from the largest series of step-up approach [7, 17]. Moreover, the total number of drainage procedures including all the steps was lower than that in previous major studies [7, 14]. These results suggest that this novel four-step approach, when applied by an experienced team, does not place subjects at greater risk of mortality and morbidity and it has the potential to improve the cost-effectiveness of currently available treatment.

With the dint of PCD, NPI and ED, about three fourth of study patients avoid open surgery and most of them successfully survived (48 of 53 patients, 91 %). No procedure-related complication was observed during the study period. It is noteworthy that patients who received open necrosectomy suffered a mortality as high as 56 %, which is significantly higher than those without operation. Our rigorous indication for surgical intervention might be responsible for that. Moreover, most patients in this series received multiple minimally invasive sessions for removal of necrosis, which is in accordance to the previous reports [7, 8].

The use of NPI had been long in our center, but the tube was routinely placed during open surgery for continuous “active” drainage in the past. In the recent years, we managed to place the “double catheterization cannula” minimal-invasively under CT guidance and therefore the use of NPI could be much more extensive. As the “double catheterization cannula” can access the necrosis either peritoneally or retroperitoneally, the drainage route can be as variable as PCD and offer not only a route for continuous lavage, but also much bigger sinus tract for draining bulk of necrosis. Moreover, NPI catheter together with other pig-tail catheters could form a “drainage system” to extend the range of continuous active drainage. Briefly, lavage fluid can be infused through one or multiple pig-tails catheter and drained out by a NPI tube as shown in Fig. 3. Although the NPI catheter is very similar to the instrument described by Raraty et al. [15], our “drainage system” combining different catheters can make full use of continuous irrigation.

Different from the well-known videoscopic assisted retroperitoneal debridement (VARD), endoscopic transgastric necrosectomy (ETN) and percutaneous endoscopic necrosectomy (PEN) [5, 6, 8] techniques, we can access the target site both peritoneally or retroperitoneally through the sinus tract constructed by the “double catheterization cannula” and perform ED. Therefore no surgical incision was needed to enter the necrosis before ED and the whole procedure could be performed under conscious condition with only topical anesthesia. As it is easy to operate with very limited impairment, ED could even be repeated on a daily basis if necessary. In contrast, the previously reported techniques including VARD, ETN or PEN, need basal anesthesia to obtain a temporary access (either incision or dilation) to the necrosis before debridement and the route were relatively rigid [5, 6]. However, similar to other minimally invasive necrosectomy, our ED also face great difficulty in removing bulk of necrosis due to the limited size of access tract. An alternative temporary trocar may offer better outlet for removing necrosis and we have started to work with that.

## Conclusion

In conclusion, our four-step approach is effective in treating IPN and add no extra risk to patients when compared with other latest step-up strategies. The two novel techniques (NPI and ED) could offer distinct clinical benefits without posing unanticipated risks inherent to the procedures and work well together to debride IPN.

## Additional files

Additional file 1: The mechanism of the “double catheterization cannula”. The “double catheterization cannula” was made of a 24–30F tube for continuous negative pressure drainage and a 12F urethral catheter for continuous infusion. (GIF 483 kb)

## Abbreviations

ACS: Abdominal compartment syndrome
APACHE: Acute Physiology and Chronic Health Evaluation
CECT: Contrast enhanced Computed Tomography
COPD: Chronic obstructive pulmonary disease
CRRT: Continuous renal replacement therapy
CT: Computed Tomography
CTSI: CT severity index
DBC: Determinant-based Classification
ED: Endoscopic necrosectomy
ETN: Endoscopic transgastric necrosectomy
ICU: Intensive care unit
IPN: Infected pancreatic necrosis
NPI: Negative pressure irrigation
ON: Operative necrosectomy
PCD: Percutaneous catheter drainage
PEN: Percutaneous endoscopic necrosectomy
RAC: Revised Atlanta classification
SOFA: Sequential Organ Failure Assessment
VARD: Videoscopic assisted retroperitoneal debridement
