# Supplementary figures and images for: Negative pressure irrigation and endoscopic necrosectomy through man-made sinus tract in infected necrotizing pancreatitis: a technical report
Source: BMC Surg. 2016 Nov 10;16:73. doi: 10.1186/s12893-016-0190-x (PMC5105240; doi:10.1186/s12893-016-0190-x)

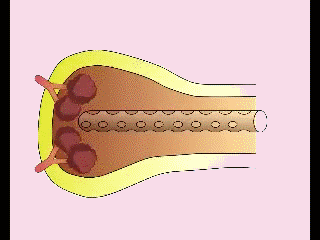

Supplement: Additional file 1: — The mechanism of the “double catheterization cannula”. The “double catheterization cannula” was made of a 24–30F tube for continuous negative pressure drainage and a 12F urethral catheter for continuous infusion. (GIF 483 kb) [file 12893_2016_190_MOESM1_ESM.gif]
